# Proton Beam Therapy in Managing Unresectable Hepatocellular Carcinoma with Bile Duct Invasion

**DOI:** 10.3390/cancers14071616

**Published:** 2022-03-23

**Authors:** Ching-Hsin Lee, An-Hsin Chen, Sheng-Ping Hung, Cheng-En Hsieh, Jeng-Hwei Tseng, Po-Jui Chen, Jen-Yu Cheng, Joseph Tung-Chieh Chang, Kun-Ming Chan, Shi-Ming Lin, Chen-Chun Lin, Wei-Ting Chen, Wan-Yu Chen, Bing-Shen Huang

**Affiliations:** 1 Proton and Radiation Therapy Center, Department of Radiation Oncology, Linkou Chang Gung Memorial Hospital and University, Taoyuan City 333423, Taiwan; b9702023@cgmh.org.tw (C.-H.L.); sphung89@cgmh.org.tw (S.-P.H.); cheng-en.hsieh@uth.tmc.edu (C.-E.H.); shkevin@cgmh.org.tw (P.-J.C.); jtchang@cgmh.org.tw (J.T.-C.C.); 2 Department of Medical Imaging and Intervention, Linkou Chang Gung Memorial Hospital and University, Taoyuan City 333423, Taiwan; angel08141@cgmh.org.tw (A.-H.C.); ma0389@cgmh.org.tw (J.-H.T.); 3 Cancer Biology and Immunology Programs, Graduate School of Biomedical Sciences, University of Texas MD Anderson UTHealth, Houston, TX 77030, USA; 4 Department of Radiation Oncology, Kaohsiung Chang Gung Memorial Hospital, Chang Gung University College of Medicine, Kaohsiung City 83301, Taiwan; york480@cgmh.org.tw; 5 Department of General Surgery, Linkou Chang Gung Memorial Hospital and University, Taoyuan City 333423, Taiwan; chankunming@cgmh.org.tw; 6 Division of Gastroenterology and Hepatology, Department of Internal Medicine, Linkou Chang Gung Memorial Hospital and University, Taoyuan City 333423, Taiwan; lsmpaicyto@cgmh.org.tw (S.-M.L.); lincc53@cgmh.org.tw (C.-C.L.); weiting@cgmh.org.tw (W.-T.C.); 7 Division of Radiation Oncology, Department of Oncology, National Taiwan University Hospital, Taipei City 100225, Taiwan; b89401088@ntu.edu.tw; 8 Cancer Research Center, National Taiwan University College of Medicine, Taipei City 100225, Taiwan; 9 Graduate Institute of Clinical Medical Science, Chang Gung University, Taoyuan City 333323, Taiwan

**Keywords:** liver cancer, hepatic malignancy, bile duct tumor thrombus, biliary tract invasion, icteric, HCC, proton therapy

## Abstract

**Simple Summary:**

Hepatocellular carcinoma with bile duct invasion is well known for its poor survival outcome, and no definite treatment guideline has been suggested. This study analyzed the treatment outcome and toxicity of proton beam therapy in managing patients with unresectable hepatocellular carcinoma with bile duct invasion. After a median follow-up of 19.9 months, proton beam therapy provided an optimal in-field control rate for these patients. The overall survival results were comparable with surgical series. This study concluded that proton beam therapy offers desirable treatment outcomes with acceptable toxicities. Based on the results, proton beam therapy provides patients with another optimal alternative for treating this notorious subtype of hepatocellular carcinoma.

**Abstract:**

Hepatocellular carcinoma (HCC) with bile duct invasion is a rare and notorious subtype of HCC. This study included patients that had unresectable HCC with bile duct invasion and proton beam therapy between November 2015 and February 2021. Twenty patients fit the inclusion criteria. The median tumor size was 6.3 cm. Nine patients (45.0%) had major vascular invasions. All included patients received the radiation dose of 72.6 gray relative biological effectiveness due to the proximity of porta hepatis and tumor. The median follow-up time was 19.9 months. The median overall survival was 19.9 months among deceased patients. The 1-year cumulative local recurrence rates were 5.3%, with only two patients developing in-field failure. The 1-year and 2-year overall survival rates were 79.4% and 53.3%. The 1-year progression-free survival was 58.9%. Four patients developed radiation-induced liver disease. The 1-year cholangitis-free survival was 55.0%. Skin toxicity was the most common acute toxicity and rarely severe. Eight patients developed ≤ grade 3 gastrointestinal ulcers. Proton beam therapy offers desirable survival outcomes for unresectable HCC patients with bile duct invasion. Optimal local tumor control could also be obtained within acceptable toxicities.

## 1. Introduction

Hepatocellular carcinoma (HCC) has been reported to be the fifth most common cancer and the second-leading cause of cancer-related death worldwide [1]. Approximately 0.5–13% of HCC patients are found with bile duct invasion [2]. A poorer prognosis has also been reported in patients with decompensated liver function and biliary tract infection-related sepsis due to local tumor progression [2,3,4]. Even in stage I and II patients, bile duct tumor thrombus (BDTT) remained a significant risk factor in overall survival (OS) and progression-free survival (PFS) [5]. Portal vein thrombosis (PVT) could be found in approximately 30% of HCC patients [6], and it was notorious for its dismal possibility to be cured [1,7]. However, the presence of BDTT was often accompanied by portal vein thrombosis (PVT), which led to a more desperate condition [8]. In a large retrospective study, the median OS of HCC patients with bile duct invasion was 4.1 months; the optimal survival was achieved in the surgical group with a median OS of 11.5 months while other treatment modalities could only obtain a 6-month median OS or less [3]. According to the European Association for the Study of the Liver (EASL) clinical practice guidelines, hepatectomy should be performed after multi-parametric composite assessment of liver function, portal hypertension, performance status, and patients’ co-morbidities [1]. Those requirements pushed most HCC patients with bile duct invasion away from the optimal treatment and resulted in an inferior prognosis.

Radiotherapy has been expanding its role in managing liver tumors because potentially ablative doses could be safely delivered using modern techniques [9]. However, radiation-induced liver disease (RILD) is still a major concern for patients undergoing photon therapy [10,11,12]. On the other hand, proton beam therapy (PBT) possesses the characteristic Bragg peak that results in no additional radiation dose beyond the target area. Studies have shown lower liver toxicities in HCC patients treated by PBT than those treated by photon therapy [13,14]. Patients with large tumors or small normal livers treated by PBT also obtained optimal local control with minimal liver toxicities [15,16]. However, with a dismal prognosis, no recommendations for treatment guidelines have been made for unresectable HCC patients with bile duct invasion [1,17]. To the best of our knowledge, there is no study focusing on the clinical outcome of PBT for managing such scenarios. Therefore, this study aimed to evaluate the clinical outcomes of PBT in patients with unresectable HCC with bile duct invasion.

## 2. Materials and Methods

### 2.1. Patients

This study adhered to the declaration of Helsinki and was approved by the local institutional review board (202100852B0). This study included patients who had unresectable HCC with bile duct invasion and excluded patients with distant metastasis. Inclusion criteria were: (1) Histologically confirmed or image-diagnosed primary HCC without distant metastasis. Image-diagnosed patients required typical presentations, including wash-in at arterial phase and wash-out at venous and delay phase on computed tomography (CT) or magnetic resonance imaging (MRI); (2) Unresectable HCC with bile duct invasion. The diagnostic image required bile duct dilatation and/or filling defect in the bile duct without bile duct wall thickening [18,19,20]; (3) Eastern Cooperative Oncology Group (ECOG) performance score ≤ 2. Between 2016 and February 2021, a total of 521 HCC patients received definitive PBT. After filtering all treated patients by using inclusion criteria, 20 patients were included in this study. Extents of bile duct invasions were reviewed by radiologists using the UEDA classification [21]. The UEDA classification included: (1) type I: BDTT located in the secondary branch of the biliary tree; (2) type II: BDTT extended to the first branch of the biliary tree; (3) type IIIa: BDTT extended to the common hepatic duct; (4) type IIIb: implanted tumor growing to the common hepatic duct; (5) type IV: dislodged BDTT within the common hepatic duct [22].

### 2.2. Treatment

Since November 2015, PBT has been administered at Chang Gung Memorial Hospital, Linkou branch, for clinical use. Proton beams were generated using a cyclotron, degraded, and then delivered using a passive scattering technique or a pencil beam scanning technique. Our pencil beam scanning technique started to be applied on HCC patients in 2017 after a system upgrade. The disposition of beam delivery took dose coverage, dose conformality, and patients’ compliances to respiratory motion management into consideration. The patients were treated using rotational gantries. Abdominal belt compression was used to reduce respiratory motions. All patients were trained to manage diaphragm motion within 1 cm. All patients were requested to fast at least 2 h before CT simulation and treatment. Simulation images—including dynamic CT, 4D-CT, and contrast-enhanced MRI—were obtained to determine the tumor motion and margin. The gross tumor volume was defined as the identifiable lesions on CT and MRI images. A clinical target volume (CTV) was contoured as the gross tumor volume plus a 5 mm margin on serial CT images used in the treatment system. Increased CTV margin or respiratory gating were applied for patients who failed to achieve the limitation for respiratory management. All treatment was performed once a day, five days a week, using a two- to three-beam arrangement with energy ranging from 70 to 230 MeV. Daily patient alignments were performed using 2D kV images for matching, and daily fluoroscopies were applied to confirm patients’ compliance to respiratory motion management.

The major protocols were 72.6 gray relative biological effectiveness (GyRBE) in 22 fractions for central tumors (≤2 cm from gastrointestinal tracts and porta hepatis) [23,24]. Dose constraints were as follows: gastrointestinal (GI) tract: Dmax (maximal dose) <65% of the total dose; spinal cord: Dmax < 39 GyRBE. In the event of CTV comprising the GI tract, dose coverage was reduced but not lowered below 60%. The mean liver dose, normal liver volume (NLV), and clinical target volume were recorded. The non-irradiated liver volume (NILV) was defined as the liver volume receiving less than 1 GyRBE.

### 2.3. Follow-Up

Patients visited the radiation oncologist on a weekly basis during treatment. Complications were assessed using the Common Terminology Criteria for Adverse Events v4.0 (CTCAE). Follow-up with patients was conducted one month after completing the treatment course and continued at 3-month intervals. During follow-up, patients were evaluated through physical examination, blood tests, and abdominal imaging studies (CT or MRI). In addition, the tumor response was examined by radiologists according to the modified Response Evaluation Criteria in Solid Tumors [25]. Local recurrences of tumor were defined as tumor recurrence in the irradiated area; hepatic recurrences indicated hepatic tumor recurrences out of irradiated fields; distant failure represented disease recurrence arising from distant metastasis. RILD was diagnosed according to patients’ symptoms, laboratory, and image examinations. Classic RILD was defined as the presence of an elevated alkaline phosphatase level (more than twice the upper limit of the normal or baseline value) and symptoms of anicteric hepatomegaly and ascites, occurring two weeks to three months after PBT. Non-classic RILD was defined as dysregulation in hepatic function with jaundice and/or an elevated serum transaminase level (a more than five-fold increase compared with the normal level) or Child–Pugh scores deterioration of more than 2 points occurring one week to three months after PBT [11,12].

### 2.4. Statistical Analysis

Actual survival and disease recurrence rates were calculated from the first day of PBT to the date of the event of interest (or censored on the last follow-up date). Survival and cholangitis-free survivals were plotted with the Kaplan–Meier method (log-rank test). Local recurrence, hepatic recurrence, and distant failure rate were estimated using cumulative incidence function. Statistical analyses were performed using commercial statistical software packages (SPSS, version 25.0, IBM Corporation, NY, USA, and R version 4.1.2 (1 November 2021) using cmprsk package [26]).

## 3. Results

**Figure 1:**
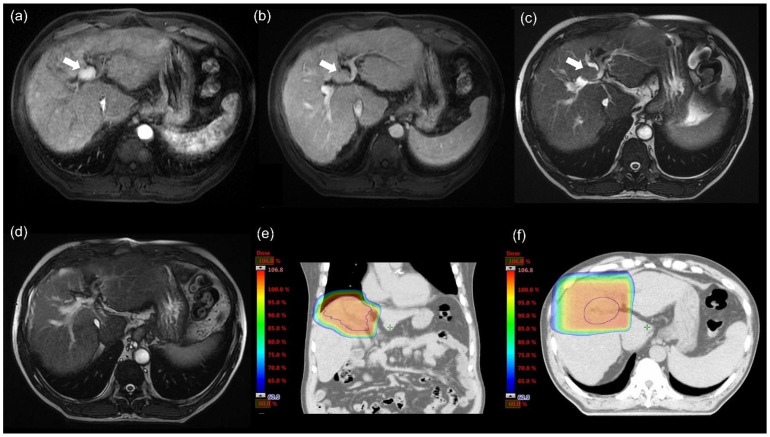
Tumor response of hepatocellular carcinoma. The tumor thrombus (white arrow) in bile duct showed washed-in in the arterial phase (**a**) and washed-out in the venous phase (**b**) of magnetic resonance imaging (MRI). (**c**) Tumor thrombus (white arrow) in bile duct could also be identified in T2 phase of MRI. (**d**) Irradiated tumor thrombus had a complete response one month after proton beam therapy (PBT). (**e**) The coronal view of the treatment plan. (**f**) The axial view of the treatment plan.

This study included 20 patients with unresectable HCC with bile duct invasion who received definitive PBT (Table 1). The median age at diagnosis was 61.5 years old, and only three patients were female. Six patients were newly diagnosed with HCC, and 14 patients had recurrent disease that were treated by other modalities before. The median follow-up time was 19.9 months (range = 3.1–64.9 months). During follow-up, 12 patients died with a median OS of 19.9 months. The median tumor size was 6.3 cm (range: 1.0–18.5 cm). One patient had nodal metastasis, while nine patients (45.0%) had major vascular thrombosis. None of the included patients were classified as UEDA type I. Five patients had UEDA type II invasion. UEDA type IIIa invasions were found in six patients, while only two patients exhibited UEDA type IIIb invasions. The remaining seven patients had UEDA type IV invasions. All UEDA type IV patients had combinations of other invasion sites (type II:1; type IIIa 6). All of the included patients received the radiation dose of 72.6 GyRBE due to the proximity of porta hepatis and tumor (Figure 1). They completed scheduled treatment within five weeks. The median clinical target volume (CTV) was 280.3 cm^3^ (range: 35.8–1852.4 cm^3^). The median NLV and NILV were 1229.3 cm^3^ (range: 694.3–1723.0 cm^3^) and 613.2 cm^3^ (range: 104.4–1034.4 cm^3^) (Table 2).

**Figure 2:**
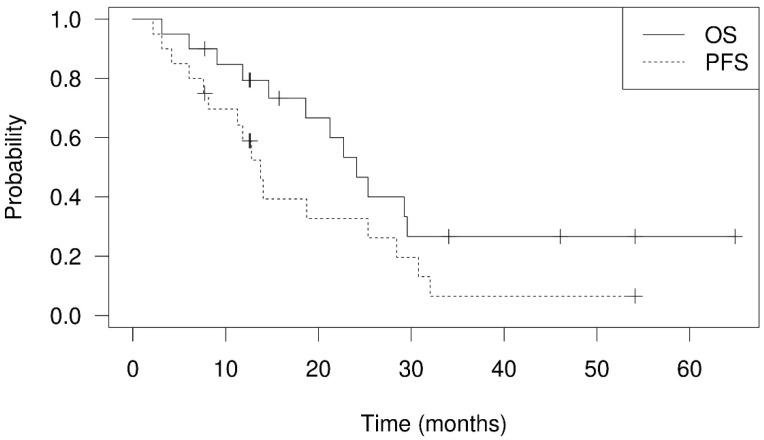
Overall survival (OS) and progression-free survival (PFS) plots of hepatocellular carcinoma patients with bile duct invasion treated with proton beam therapy. The 1-year and 2-year overall survival rate were 79.4% and 53.3%. The 1-year progression free survival was 58.9%.

**Figure 3:**
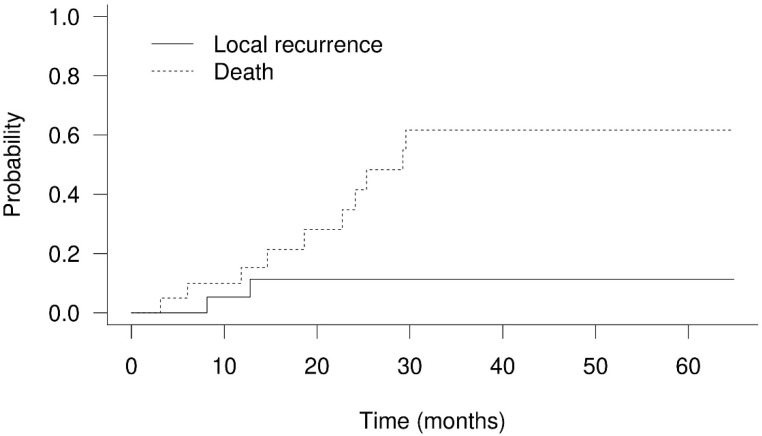
Cumulative local recurrence and death rate of hepatocellular carcinoma with bile duct invasion treated with proton beam therapy. The 1-year cumulative local recurrence rate was 5.3%.

**Figure 4:**
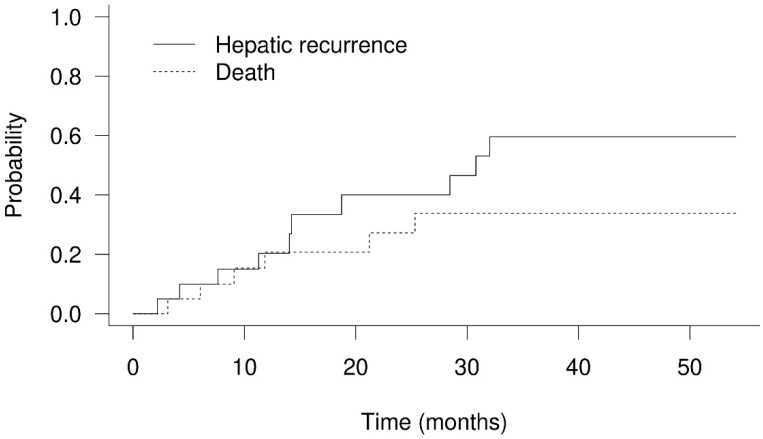
Cumulative hepatic recurrence and death rate of hepatocellular carcinoma with bile duct invasion treated with proton beam therapy. The 1-year cumulative hepatic recurrence rate was 20.4%.

**Figure 5:**
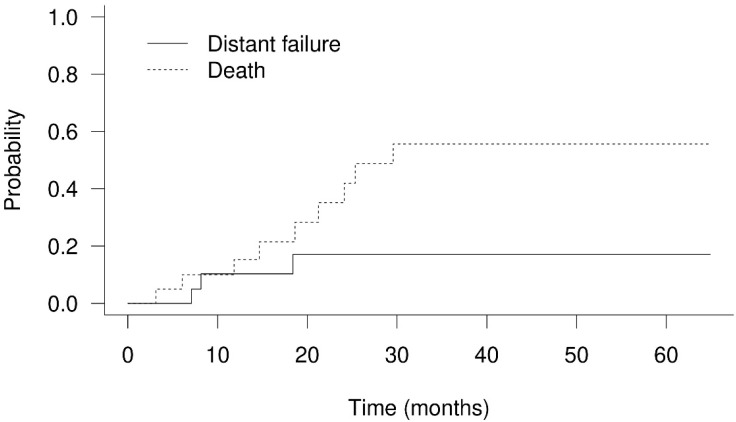
Cumulative distant failure and death rate of hepatocellular carcinoma with bile duct invasion treated with proton beam therapy. The 1-year cumulative distant failure rate was 10.3%.

The 1-year and 2-year OS rates were 79.4% and 53.3% (Figure 2), respectively. Twelve patients died between 3.1 months and 29.6 months after PBT. There were only two patients who had in-field failure in this study. One of them died of GI bleeding caused by tumor invasion to the duodenum, confirmed by endoscopic examination. The area of progressing tumor was delivered with a 60% prescribed dose according to GI constraints in the treatment protocol. Another patient died of tumor progression-related obstructive jaundice. Five patients died of infection episodes, and two of them were related to cholangitis. One patient died of immunotherapy-related hepatitis. Through telephone follow-up, two patients died of cancer-related causes, and two patients died of unknown reasons. The 1-year progression-free survival was 58.9%. The 1-year cumulative local recurrence, hepatic recurrence, and distant failure rates were 5.3% (Figure 3), 20.4% (Figure 4), and 1.0% (Figure 5), respectively. Survival and disease control failed to show significant differences among different degrees of bile duct invasion due to the small sample size. Upon first post-PBT imaging evaluation, patient numbers with complete response (CR), partial response (PR), and stable disease (SD) in the irradiated area were 4, 13, and 3 (20%, 65%, and 15%, respectively). In the latest imaging evaluation, 14 patients (70%) had CR in the irradiated area. The median time to CR was 4.8 months (range: 2.1–8.3 months). Four patients (20%) had partial responses, while two patients experienced in-field progression 8.2 months and 12.8 after PBT. No statistical differences in survival and disease control were found between different stages or extents of bile duct invasion. Increased serum AFP levels were noted in the nine patients before PBT. The median serum AFP level before PBT decreased from 447.2 ng/mL (range: 23.0–440,589.0 ng/mL) to 21.5 ng/mL (range: 4.5–10,075.4 ng/mL) one month after PBT (Table 1 and Supplementary Table S1).

All of the included patients were found with bile duct invasion. Seven patients received bile duct drainage before PBT, and only one of them had no further intervention after PBT. Around half of UEDA type IIIa, IIIb, and IV patients required bile duct drainage before PBT (type IIIa: 3 patients; type IIIb: 1; type IV: 3). Nine patients received intervention for bile duct drainage 1.4–21.7 months after PBT. Above half of UEDA type IIIa and IV patients required bile duct drainage after PBT (type IIIa: 4; type IV: 4); one patient with UEDA type II invasion had bile duct drainage after PBT. Eight patients had cholangitis that required antibiotic treatment, and most of them were UEDA type IIIa and IV patients. The first cholangitis episodes occurred within 1.6–21.6 months after PBT, and four occurred within three months after PBT. Among eight patients, two of them died of cholangitis-related infection episodes around two years after PBT, while one of them died of obstructive jaundice caused by tumor progression. The 1-year cholangitis-free survival was 55.0%.

Acute toxicities involving the skin were observed in 13 patients (65.0%). One patient developed grade 2 toxicity, and one developed grade 3 toxicity. Regarding GI toxicities, no patient developed esophagitis or colitis, but grade 2 and grade 3 gastroduodenal ulcers were found on three and five patients, respectively. Four patients developed RILD. Two of them had non-classic RILD, while the other two patients had both classic and non-classic RILD. Their NILV ranged from 269.3 cm^3^ to 981.8 cm^3^. One of them died two months after PBT without medical records in the institution. Therefore, the possibility of RILD-related death could not be ruled out. The patient initially had an UEDA type IV bile duct invasion, Child–Pugh score 7, and NILV 269.3 cm^3^. Among patients who recovered from RILD, one initially had an UEDA type IV bile duct invasion, Child–Pugh score 8, and NILV 981.8 cm^3^. The other two patients were in Child–Pugh class A, with NILV over 350 cm^3^ (398.2 and 653.1 cm^3^), and UEDA type II and IIIa bile duct invasion. No statistical differences in RILD rate were found between different stages or extents of bile duct invasion. More details about included patients were listed in Supplementary Table S1.

## 4. Discussion

The optimal treatment strategy for HCC patients with bile duct invasion has not been developed [1,17]. Although surgical excision possesses the best survival outcome in historical data, most patients are not eligible for surgery due to compromised liver function, insufficient liver remnant, and hepatic hilar involvement [1,3,4,27]. In a retrospective study, patients receiving non-surgical treatment were reported to have a median OS ranging from 1.6 to 6.0 months [3]. More recent studies showed that trans-arterial chemo-embolization (TACE) offers a median OS of about 3 months and 12.2 months for recurrent and newly diagnosed patients, respectively [28,29]. As a less discussed modality, percutaneous endobiliary radiofrequency ablation (RFA) and stent provided a 6-month median OS [30].

In this study, the median follow-up time was 19.9 months, and the median OS was 19.9 months among deceased patients. The OS data revealed a favorable prognosis compared to the historical data with treatment modalities of TACE, RFA, chemotherapy, or other conservative treatment modalities [3,28,29,30]. The median and 1-year OS in this study were comparable to surgical series with imaging diagnoses of HCC with bile duct invasion. The surgical treatment served as the primary treatment for newly diagnosed HCC; on the other hand, the majority of patients in this study had recurrent disease and only six patients were newly diagnosed. Those studies revealed 60.5–75.0% 1-year OS and 16.6–19 months median OS in patients treated by surgical excisions [31,32,33]. Patients with tumor thrombus in major vessels accounted for nearly half of the population in this study; this kind of patient only accounted for 25.0% or less in surgical series [31,32,33]. UEDA et al. found that type II and IIIa had a relatively poorer prognosis than other subtypes [21]. However, results from the present study and surgical series could not support this conclusion due to the lack of adequate analysis. The 1-year PFS of 58.9% in the current study was also similar to the 1-year cumulative recurrence rate of 49.2% reported in another surgical cohort [34]. The complication rates were higher in this study compared to the surgical series. However, the post-operative mortality rate ranged from 1.8% to 8%, while no patient in this study deceased within treatment or one month after PBT (Table 3).

Aside from survival results, tumor response rate and local recurrence rate also achieved desirable goals in this study. Upon first post-treatment imaging evaluation, historical data of TACE provided only 2–4%, 21–38%, and 24–37% in CR, PR, and progressive disease (PD) rates separately [3,28,29,35]. The current study showed that the first post-treatment imaging evaluation’s CR, PR, and SD rates were 20%, 65%, and 15%, respectively. The response rate sustained a favorable outcome in the latest imaging evaluation with a 70% complete response rate. With only two patients developing in-field failure within one year after PBT, the 1-year cumulative local recurrence rate was 5.3%. This result was similar to previously reported 90% local control rate in HCC patients treated by PBT [14,15,16,36,37]. The optimal local control within irradiated area was achieved by delivering ablative radiation doses to the liver tumor while preserving adequate liver function. A slightly higher RILD rate of 20% was reported in this study compared to 11.8–14% in other studies that utilized PBT to treat HCC patients [13,38]. Regarding liver tolerance to PBT, Mizumoto et al. suggested the optimal cut-offs for V0, V10, V20, and V30 were 30%, 20%, 26%, and 18%, respectively [39]. V30 less than 25% was taken as an important predictor of RILD for patients with ICG-R15 of 20% to 49.9% by Kawashima et al. [40]. None of the included patients could fully fit the suggested cut-off for normal liver constraints, but only four patients developed RILD. Therefore, the suggested cut-off might not be fully practical in our cases. Aside from doses to liver volume, Toramatsu et al. suggested tumor volume as an indicator for developing RILD when comparing photon and proton beam therapy [41]. However, this study had a worse performance in RILD when compared with studies regarding large liver tumors [15] and small liver volume [16], even with similar median tumor size [16] or dosimetric data. The presence of bile duct invasion could be considered the factor affecting performances in RILD in this study. This study showed that PBT provided optimal tumor response and low local recurrence rate even with unresectable HCC combining bile duct invasion. However, liver constraints should be evaluated carefully regarding the influence of bile duct invasion.

The poor prognosis of HCC patients with bile duct invasion results from decompensated liver function and biliary tract infection-related sepsis due to local tumor progression [2,3,4]. Most patients in this study obtained good in-field control and mitigated risks mentioned above. However, cholangitis had also been reported as a rare complication of radiotherapy mentioned by a few case reports [42,43]. Hence, radiotherapy-induced cholangitis should be noticed due to the increasing number of patients receiving ablative doses. Meanwhile, toxicities such as dermatitis and GI ulcerations still existed. Dermatitis has been recognized as one of the most common toxicities when liver tumors were treated using PBT [15,36,44]. Dermatitis might be related to overlapping fields and a lack of skin-sparing effect in PBT. Therefore, the angle of treatment fields should be carefully evaluated, considering aspects of tumor size, NILV, as well as skin toxicities. GI ulceration was the second common toxicity in this study. HCC with bile duct invasions were mostly close to GI tracts. Therefore, the prevalence of GI toxicities was higher than other PBT studies that included patients with liver tumors both close to and away from GI tracts [36].

This study still poses certain limitations. First of all, the nature of retrospective studies is prone to have selection bias. Second, this single-institutional study only provided a small sample size and a limited follow-up period. Hence, the data should be cautiously interpreted. Finally, the small sample size failed to offer statistical differences in survival, disease control, and complication rate between stages and extents of bile duct invasion. Therefore, further prospective studies should be conducted to confirm and expand current results.

## 5. Conclusions

Unresectable HCC with bile duct invasion could be effectively treated by PBT because of the successful delivery of the ablative dose. Proton beam therapy extended the treatment of choice for patients who could not receive surgical treatment with minimal post-treatment mortality risks. Liver constraints should be carefully amended considering influences of bile duct invasion. Physicians should also beware of GI and skin toxicities when planning PBT. PBT offers an optimal in-field tumor control and a favorable OS for the notorious and rare subtypes of unresectable HCC involving bile duct invasions with acceptable toxicities.

## Figures and Tables

**Table 1:** Patient characteristics (N = 20).

Variable	N	%
Median age (years)	61.5 (42–83)	
Male sex	17	85.0
Performance status		
0	7	35.0
1	13	65.0
Viral hepatitis		
HBV	13	65.0
HCV	3	15.0
None	4	20.0
Child–Pugh score		
5	9	45.0
6	7	35.0
7	2	10.0
8	1	5.0
9	1	5.0
Baseline laboratory exam	Median (range)	
Albumin (g/dL)	4.0 (2.7–4.7)	
Total bilirubin (mg/dL)	1.7 (0.5–8.6)	
INR	1.2 (1.0–1.8)	
AST (U/L)	70.0 (37.0–202.0)	
ALT (U/L)	67.0 (17.0–368.0)	
Alk-P (U/L)	142 (62–349)	
AFP (ng/mL)	447.2 (23.0–440,589.0)	
Previous treatment	N	%
Surgery	2	10.0
RFA	7	35.0
TACE	10	50.0
HAIC	2	10.0
Sorafenib	3	15.0
Immunotherapy	1	5.0
None	5	25.0
Combined treatment during PBT		
Sorafenib	3	15.0
Immunotherapy	1	5.0
None	16	80.0
Median sum of tumor diameter	6.3 (1–18.5) cm	
<5.0 cm	7	35.0
5.0–9.9 cm	6	30.0
≥10.0 cm	7	35.0
Numbers of tumor		
Single	9	45.0
Multiple	11	55.0
Vascular thrombosis		
Segmental portal vein	5	25
Main portal vein	9	45
TNM stage ^a^		
IA	1	5.0
IB	4	20.0
II	4	20.0
IIIA	2	10.0
IIIB	8	40.0
IVA	1	5.0
BCLC		
0	1	5.0
A	4	20.0
B	2	10.0
C	13	65.0
UEDA classification		
II	5	20.0
IIIa	6	35.0
IIIb	2	10.0
IV (combined with II/IIIa)	7 (1/6)	35.0 (5.0/30.0)

HBV: hepatitis B virus; HCV: hepatitis C virus; INR: international normalized ratio; AST: aspartate transaminase; ALT: alanine transaminase; Alk-P: alkaline-phosphatase; AFP: alpha-fetoprotein; RFA: radiofrequency ablation; TACE: transarterial chemoembolization; HAIC: hepatic arterial infusion chemotherapy; PBT: proton beam therapy; BCLC: Barcelona Clinic Liver Cancer. ^a^ TNM stage was using the American Joint Committee on Cancer Eighth edition.

**Table 2:** Summary of dose–volume analysis.

Variable	Median (Range)
CTV (cm^3^)	280.3 (35.8–1852.4)
NLV (cm^3^)	1229.3 (694.3–1723.0)
Mean dose (GyRBE)	16.8 (11.5–28.0)
NILV (cm^3^)	613.2 (104.4–1034.4)
V10 (%)	35.9 (27.9–56.4)
(cm^3^)	396.2 (240.1–813.0)
V20 (%)	30.7 (23.6–50.2)
(cm^3^)	345.8 (197.9–723.7)
V30 (%)	25.7 (17.7–42.8)
(cm^3^)	291.5 (162.5–618.2)
V40 (%)	20.9 (9.5–37.6)
(cm^3^)	242.6 (128.4–544.3)

CTV: clinical tumor volume; NLV: normal liver volume; NILV: non-irradiated liver volume; V10, V20, V30, V40: percentage of normal liver volume that received ≥ 10 GyRBE, ≥20 GyRBE, ≥30 GyRBE, and ≥40 GyRBE.

**Table 3:** Summary of surgical series and this study.

Study	Patient No.	Child–Pugh Class	Total Bilirubin (mg/dL)	Tumor Size	Multiple Tumor	TNM *	UEDA Classification	Major Vascular Invasion	Treatment	Overall Survival	Toxicity
2018, Xinwei Yang, et al. [33]	107	N/A	Median: 3.57	<5 cm: 49; ≥5 cm: 58	Yes: 22; No: 85	I: 16; II: 24; III: 62; IV: 5	N/A	Yes: 13; No: 94	Hepatectomy: 107	Median: 16.6 months; 1-y: 60.5%; 3-y: 20.1%, 5-y: 12.0%	Mortality:2; pleural effusions: 5; hemobilia:3; biliary tract infection:1; bile leakage: 1; upper gastrointestinal ulcer bleeding: 1; thoracic epidural hematoma: 1; infection at the incision site:2
2019, Zhichuan Lin, et al. [32]	25	N/A	≤11.7: 16; >11.7:9	<5 cm: 13; ≥5 cm: 12	Yes: 2; No: 23	N/A	I: 2; II: 2; III: 21	Yes: 5; No: 20	Hepatectomy: 25 (radical resection)	Median: 19 months; 1-y: 68.0%; 3-y: 32.0%, 5-y: 24.0%	Mortality:2; gastrointestinal ulcer bleeding: 2; subphrenic effusion: 3; pulmonary infection: 3
2020, Qiyu Chi, et al. [31]	25	A: 20; B: 5	N/A	6.97 ± 3.45 cm	Yes: 4; No: 21	I: 8; II: 11; III: 6	I: 2; II: 8; III:15	Yes: 5; No 20	Hepatectomy: 25	1-y: 75.0%; 3-y: 38.7%, 5-y: 17.7%	Perioperative mortality: 4%
This study	20	A: 16; B: 4	Median: 1.7; ≤1: 6; >1:13	<5 cm: 10; ≥5 cm: 10	Yes: 11; No: 9	I: 5; II: 4; IIIa: 2; IIIb: 8l; IVa: 1	II: 4; IIIa: 7; IIIb: 2; IV: 7	Yes: 9; No 11	Proton beam therapy	Median: 19 months; 1-y: 79.4%; 2-y: 46.6%	Dermatitis: 13 (65%); gastrointestinal ulcer: 8 (40%); RILD: 4 (20%)

N/A: not applicable; y: year; ISGLS: International Study Group of Liver Surgery; RILD: radiation-induced liver disease. * TNM stage was using the American Joint Committee on Cancer Seventh edition for comparisons with surgical series.

## Data Availability

The statistical datasets and codes used and/or analyzed in the current study are available from the corresponding author (beanson@cgmh.org.tw) on reasonable request.

## Supplementary Materials

The following supporting information can be downloaded at: https://www.mdpi.com/article/10.3390/cancers14071616/s1, Table S1: Details of patients’ data.

## References

[1] European Association for the Study of the Liver (2018). EASL clinical practice guidelines: Management of hepatocellular carcinoma. J. Hepatol..

[2] Huang J.F., Wang L.Y., Lin Z.Y., Chen S.C., Hsieh M.Y., Chuang W.L., Yu M.L., Lu S.N., Wang J.H., Yeung K.W. (2002). Incidence and clinical outcome of icteric type hepatocellular carcinoma. J. Gastroenterol. Hepatol..

[3] An J., Lee K.S., Kim K.M., Park D.H., Lee S.S., Lee D., Shim J.H., Lim Y.-S., Lee H.C., Chung Y.-H. (2017). Clinical features and outcomes of patients with hepatocellular carcinoma complicated with bile duct invasion. Clin. Mol. Hepatol..

[4] Suh Y.-G., Do Young Kim K.-H.H., Seong J. (2014). Effective biliary drainage and proper treatment improve outcomes of hepatocellular carcinoma with obstructive jaundice. Gut Liver.

[5] Wu J.-Y., Sun J.-X., Wu J.-Y., Huang X.-X., Bai Y.-N., Wei Y.-G., Zhang Z.-B., Zhou J.-Y., Cheng S.-Q., Yan M.-L. (2022). Impact of bile duct tumor thrombus on the long-term surgical outcomes of hepatocellular carcinoma patients: A propensity score matching analysis. Ann. Surg. Oncol..

[6] Lu J., Zhang X.-P., Zhong B.-Y., Lau W.Y., Madoff D.C., Davidson J.C., Qi X., Cheng S.-Q., Teng G.-J. (2019). Management of patients with hepatocellular carcinoma and portal vein tumour thrombosis: Comparing east and west. Lancet Gastroenterol. Hepatol..

[7] Marrero J.A., Kulik L.M., Sirlin C.B., Zhu A.X., Finn R.S., Abecassis M.M., Roberts L.R., Heimbach J.K. (2018). Diagnosis, Staging, and Management of Hepatocellular Carcinoma: 2018 Practice Guidance by the American Association for the Study of Liver Diseases. Hepatology.

[8] Kasai Y., Hatano E., Seo S., Taura K., Yasuchika K., Uemoto S. (2015). Hepatocellular carcinoma with bile duct tumor thrombus: Surgical outcomes and the prognostic impact of concomitant major vascular invasion. World J. Surg..

[9] Klein J., Dawson L.A. (2013). Hepatocellular carcinoma radiation therapy: Review of evidence and future opportunities. Int. J. Radiat. Oncol. Biol. Phys..

[10] Jung J., Kim H., Yoon S.M., Cho B., Kim Y.J., Kwak J., Kim J.H. (2018). Targeting Accuracy of Image-Guided Stereotactic Body Radiation Therapy for Hepatocellular Carcinoma in Real-Life Clinical Practice: In Vivo Assessment Using Hepatic Parenchymal Changes on Gd-EOB-DTPA–Enhanced Magnetic Resonance Images. Int. J. Radiat. Oncol. Biol. Phys..

[11] Kim J., Jung Y. (2017). Radiation-induced liver disease: Current understanding and future perspectives. Exp. Mol. Med..

[12] Pan C.C., Kavanagh B.D., Dawson L.A., Li X.A., Das S.K., Miften M., Haken R.K.T. (2010). Radiation-Associated Liver Injury. Int. J. Radiat. Oncol. Biol. Phys..

[13] Cheng J., Liu C., Wang Y., Hsu H., Huang E., Huang T., Lee C., Hung S., Huang B. (2020). Proton versus photon radiotherapy for primary hepatocellular carcinoma: A propensity-matched analysis. Radiat. Oncol..

[14] Sanford N.N., Pursley J., Noe B., Yeap B.Y., Goyal L., Clark J.W., Allen J.N., Blaszkowsky L.S., Ryan D.P., Ferrone C.R. (2019). Protons versus Photons for Unresectable Hepatocellular Carcinoma: Liver Decompensation and Overall Survival. Int. J. Radiat. Oncol. Biol. Phys..

[15] Sugahara S., Oshiro Y., Nakayama H., Fukuda K., Mizumoto M., Abei M., Shoda J., Matsuzaki Y., Thono E., Tokita M. (2010). Proton Beam Therapy for Large Hepatocellular Carcinoma. Int. J. Radiat. Oncol. Biol. Phys..

[16] Lee C.-H., Hung S.-P., Hong J.-H., Chang J.T.-C., Tsang N.-M., Chan K.-M., Tseng J.-H., Huang S.-C., Lin S.-M., Lien J.-M. (2018). How small is too small? New liver constraint is needed—Proton therapy of hepatocellular carcinoma patients with small normal liver. PLoS ONE.

[17] Yang J.D., Hainaut P., Gores G.J., Amadou A., Plymoth A., Roberts L.R. (2019). A global view of hepatocellular carcinoma: Trends, risk, prevention and management. Nat. Rev. Gastroenterol. Hepatol..

[18] Zhou X., Wang J., Tang M., Huang M., Xu L., Peng Z., Li Z.-P., Feng S.-T. (2020). Hepatocellular carcinoma with hilar bile duct tumor thrombus versus hilar Cholangiocarcinoma on enhanced computed tomography: A diagnostic challenge. BMC Cancer.

[19] Long X.-Y., Li Y.-X., Wu W., Li L., Cao J. (2010). Diagnosis of bile duct hepatocellular carcinoma thrombus without obvious intrahepatic mass. World J. Gastroenterol. WJG.

[20] Liu Q.-Y., Huang S.-Q., Chen J.-Y., Li H.-G., Gao M., Liu C., Liang B.-L. (2010). Small hepatocellular carcinoma with bile duct tumor thrombi: CT and MRI findings. Abdom. Imaging.

[21] UEDA M., Takeuchi T., Takayasu T., Takahashi K., Okamoto S., Tanaka A., Morimoto T., Mori K., Yamaoka Y. (1994). Classification and surgical treatment of hepatocellular carcinoma (HCC) with bile duct thrombi. Hepatogastroenterology.

[22] Hu X.-G., Mao W., Hong S.Y., Kim B.-W., Xu W.-G., Wang H.-J. (2016). Surgical treatment for hepatocellular carcinoma with bile duct invasion. Ann. Surg. Treat. Res..

[23] Nakayama H., Sugahara S., Fukuda K., Abei M., Shoda J., Sakurai H., Tsuboi K., Matsuzaki Y., Tokuuye K. (2011). Proton beam therapy for hepatocellular carcinoma located adjacent to the alimentary tract. Int. J. Radiat. Oncol. Biol. Phys..

[24] Mizumoto M., Okumura T., Hashimoto T., Fukuda K., Oshiro Y., Fukumitsu N., Abei M., Kawaguchi A., Hayashi Y., Ookawa A. (2011). Proton beam therapy for hepatocellular carcinoma: A comparison of three treatment protocols. Int. J. Radiat. Oncol. Biol. Phys..

[25] Lencioni R., Llovet J.M. (2010). Modified RECIST (mRECIST) Assessment for Hepatocellular Carcinoma. Semin. Liver Dis..

[26] Gray B. (2022). cmprsk: Subdistribution Analysis of Competing Risks. http://CRAN.R-project.org/package=cmprsk.

[27] Qin L.-X., Tang Z.-Y. (2003). Hepatocellular carcinoma with obstructive jaundice: Diagnosis, treatment and prognosis. World J. Gastroenterol..

[28] Park J., Kim H.-C., Lee J.-H., Cho E., Kim M., Hur S., Jae H.J., Lee M., Chung J.W. (2018). Chemoembolisation for hepatocellular carcinoma with bile duct invasion: Is preprocedural biliary drainage mandatory?. Eur. Radiol..

[29] Choi J.W., Chung J.W., Cho Y.K., Kim Y.J., Yoon J.-H., Kim H.-C., Jae H.J. (2015). Transarterial chemoembolization for hepatocellular carcinomas with central bile duct invasion: Safety, prognosis, and predictive factors. Cardiovasc. Interv. Radiol..

[30] Cui W., Xu R., Wang Y., Shi F., Li J., Chen X. (2020). Percutaneous endobiliary radiofrequency ablation and stents in management of hepatocellular carcinoma with bile duct tumor thrombus: Initial single-institution experience. Asia Pac. J. Clin. Oncol..

[31] Chi Q., Shi Z., Zhang Z., Zhang X., Zhang L., Weng S. (2020). Outcomes of resection for hepatocellular carcinoma with macroscopic bile duct tumour thrombus: A propensity score matched study. Oncol. Lett..

[32] Lin Z., Han M., Zhou Z. (2019). Prognosis for patients with hepatocellular carcinoma (HCC) with bile duct tumor thrombus (BDTT) after surgical treatment. Biosci. Trends.

[33] Yang X., Qiu Z., Ran R., Cui L., Luo X., Wu M., Tan W.F., Jiang X. (2018). Prognostic importance of bile duct invasion in surgical resection with curative intent for hepatocellular carcinoma using PSM analysis. Oncol. Lett..

[34] Kim D.-S., Kim B.-W., Hatano E., Hwang S., Hasegawa K., Kudo A., Ariizumi S., Kaibori M., Fukumoto T., Baba H. (2020). Surgical Outcomes of Hepatocellular Carcinoma With Bile Duct Tumor Thrombus: A Korea–Japan Multicenter Study. Ann. Surg..

[35] Yang K., Sung P.S., Oh J.S., Chun H.J., Jang J.W., Bae S.H., Choi J.Y., Yoon S.K. (2018). Transarterial Chemolipiodolization for Hepatocellular Carcinoma with Central Bile Duct Invasion Causing Conjugated Hyperbilirubinemia: Safety and Prognostic Factors for Survival. J. Liver Cancer.

[36] Hong T.S., Wo J.Y., Yeap B.Y., Ben-Josef E., McDonnell E.I., Blaszkowsky L.S., Kwak E.L., Allen J.N., Clark J.W., Goyal L. (2016). Multi-Institutional Phase II Study of High-Dose Hypofractionated Proton Beam Therapy in Patients With Localized, Unresectable Hepatocellular Carcinoma and Intrahepatic Cholangiocarcinoma. J. Clin. Oncol..

[37] Yoo G.S., Yu J.I., Cho S., Jung S.H., Han Y., Park S., Oh Y., Lee B., Park H.C., Lim D.H. (2020). Comparison of clinical outcomes between passive scattering versus pencil-beam scanning proton beam therapy for hepatocellular carcinoma. Radiother. Oncol..

[38] Hsieh C.-E., Venkatesulu B.P., Lee C.-H., Hung S.-P., Wong P.-F., Aithala S.P., Kim B.K., Rao A., Chang J.T.-C., Tsang N.-M. (2019). Predictors of radiation-induced liver disease in eastern and western patients with hepatocellular carcinoma undergoing proton beam therapy. Int. J. Radiat. Oncol. Biol. Phys..

[39] Mizumoto M., Okumura T., Hashimoto T., Fukuda K., Oshiro Y., Fukumitsu N., Abei M., Kawaguchi A., Hayashi Y., Ohkawa A. (2012). Evaluation of Liver Function After Proton Beam Therapy for Hepatocellular Carcinoma. Int. J. Radiat. Oncol. Biol. Phys..

[40] Kawashima M., Kohno R., Nakachi K., Nishio T., Mitsunaga S., Ikeda M., Konishi M., Takahashi S., Gotohda N., Arahira S. (2011). Dose–volume histogram analysis of the safety of proton beam therapy for unresectable hepatocellular carcinoma. Int. J. Radiat. Oncol. Biol. Phys..

[41] Toramatsu C., Katoh N., Shimizu S., Nihongi H., Matsuura T., Takao S., Miyamoto N., Suzuki R., Sutherland K., Kinoshita R. (2013). What is the appropriate size criterion for proton radiotherapy for hepatocellular carcinoma? A dosimetric comparison of spot-scanning proton therapy versus intensity-modulated radiation therapy. Radiat. Oncol..

[42] Chandrasekhara K.L., Iyer S.K. (1984). Obstructive jaundice due to radiation-induced hepatic duct stricture. Am. J. Med..

[43] Gorea G., Demy M., Van Nhieu J.T., Tigori J., Aubé C., Cherqui D., Oberti F., Caroli-Bosc F.-X., Calès P. (2010). Radiation-induced cholangitis with hepatocellular carcinoma. Gastroentérol. Clin. Biol..

[44] Hung S.-P., Huang B.-S., Hsieh C.-E., Lee C.-H., Tsang N.-M., Chang J.T.-C., Chen J.-S., Chou W.-C., Tseng J.-H., Hong J.-H. (2020). Clinical Outcomes of Patients With Unresectable Cholangiocarcinoma Treated With Proton Beam Therapy. Am. J. Clin. Oncol..

